# Depression as a psychosocial consequence of occupational injury in the US working population: findings from the medical expenditure panel survey

**DOI:** 10.1186/1471-2458-13-303

**Published:** 2013-04-05

**Authors:** Jaeyoung Kim

**Affiliations:** 1 Department of Preventive Medicine, College of Medicine, Keimyung University, Daegu, South Korea

**Keywords:** Occupational injury, Non-occupational injury, Depression, Psychosocial consequences, Medical expenditure panel survey

## Abstract

**Background:**

Empirical evidence describing the psychosocial consequences of occupational injury is still limited. The effect of occupational injury on depression might pose unique challenges in workers compared with other kinds of injury. This study aimed to assess the differential impact of workplace injury compared with non-workplace injury on depression over time, and to identify the potential risk factors associated with post-injury depression in the US working population.

**Methods:**

Using pooled panel data from the Medical Expenditure Panel Survey 2000–2006, a total of 35,155 workers aged 18–64 years who had been followed for about 18 months in each panel were analyzed. Injuries in the 4–5 months before baseline, and subsequent depression incidence during follow-up, were identified using ICD-9 codes for the medical conditions captured in personal interviews. A discrete time-proportional odds model was used.

**Results:**

A total of 5.5% of workers with occupational injury at baseline reported depression at follow-up, compared with 4.7% of workers with non-occupational injury and 3.1% of workers without injuries. Those with occupational injuries had more severe injuries and required longer treatment, compared with those with non-occupational injuries. Only 39% of workers with workplace injuries were paid Workers’ Compensation (WC). The association between injury and depression appeared to be stronger for workplace injury, and the adjusted odds ratio for depression was 1.72 for those with occupational injury (95% CI: 1.27–2.32), and 1.36 for those with non-occupational injury (95% CI: 1.07–1.65) compared with the no-injury group, after controlling for relevant covariates. Occupational injury was associated with higher odds of developing depression over time. WC as a source of medical payment was associated with 33% higher odds of developing depression (95% CI: 1.01–1.74). Part-time work, shorter job tenure, and long working hours were independently associated with post-injury depression risk.

**Conclusions:**

Workers with occupational injury were more likely to become depressed than those with non-occupational injury. The psychosocial consequences of occupational injury, including depression, deserve further exploration to adequately support those injured at work. This finding also emphasizes a need for early intervention to reduce the burden of depression associated with occupational injury.

## Background

Although it has been reported that traumatic injury, as a stressful life event, can lead to depression [1-3], little is known about the extent to which the work-relatedness of an injury predicts subsequent depression. Evidence suggests that those injured at work, compared with those injured elsewhere, suffer a wider range of mental health consequences, including stress, anxiety and depression [4-6]. Several factors may influence this response, including resulting disability, reduced earnings, the financial burden of treatment, difficulty in returning to work, and withdrawal from the labor market after injury [5,7,8]. Additional distress from dealing with litigation may play a unique role in explaining why occupational injury may contribute to depression more than non-occupational injury.

There has been little investigation of the specific effects of occupational injury on depression, because most studies have focused either on depression after traumatic, non-occupational injury in a clinical setting, or on physical disability after an occupational injury. Only a few researchers [5,6,9] have discussed depression as a consequence of occupational injury, separate from non-occupational injury. In addition, there is a lack of epidemiologic evidence about the effect that the work-relatedness of injury has on subsequent depression among the working population, because study populations have come from local emergency rooms or from those claiming Workers’ Compensation (WC). Researchers have not specifically investigated the differential effects on mental health outcomes of occupational *vs*. non-occupational injury.

This study investigated the impact of occupational and non-occupational injury on the incidence of depression in a longitudinal study in a representative sample of the US working population. Specific objectives were to examine whether occupational injury had a differential impact on subsequent depression, and which factors may account for any observed differences in depression risk between occupational and non-occupational injuries.

## Methods

### Data source

Data were extracted from the 2000–2006 Medical Expenditure Panel Survey (MEPS), a nationally representative household survey of the US population, primarily designed to obtain national estimates of healthcare use, expenditure, and health insurance cover. Data were collected through five sets of in-person interviews in each panel at 4–5-month intervals over 2.5 years. These five interviews correspond to five rounds of data per panel, and each panel produced data on more than 15,000 individuals. Respondents reported on health service use related to their health condition, any physical and mental health problems, and loss of work or school days as a result of illness. Information on each condition was recorded *verbatim* and later coded by professional coders into appropriate International Classification of Diseases, 9th Revision (ICD-9) codes. The overall response rate across panels have generally ranged from 65% to 71%, with individual follow-up response rates at over 90% [10].

This study was exempt from the requirement for subject consent under category 4 (research of existing data publicly available) by the Harvard School of Public Health Human Subjects Committee (IRB).

### Study population

**Figure 1:**
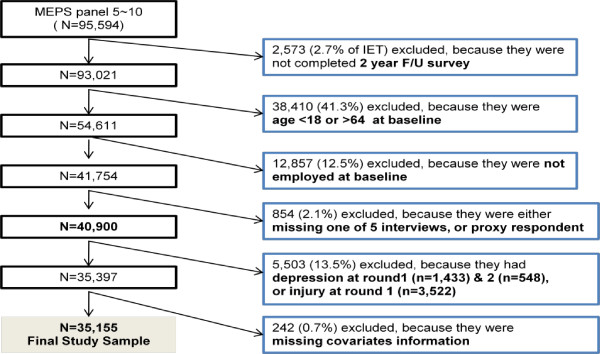
Selection process for the final study sample.

Figure 1 illustrates the exclusion process for the final analytic sample. The longitudinal panel was constructed using the household respondents’ files for each year, later merged with the files on medical conditions and job information. Six constructed MEPS panels were pooled. Panel 5 began its interviews in 2000 and Panel 10 ended its interviews in 2006. The pooled data yielded an initial eligible total (IET) of 95,594 respondents. The baseline was set as Round 2, and the information at Round 1 was used as an indicator of a previous history of depression or other comorbidity.

Individuals were excluded from the sample if they met any of the following six criteria: 1) they did not complete the 2-year survey in each panel due to death, departure from the United States, institutionalization, or military service (n = 2,573; 2.7% of IET); 2) they were not eligible for all five rounds (n = 706); 3) they had a proxy interview (n = 148); 4) they were aged under 18 or over 65 (n = 38,410); 5) they were unemployed at baseline (n = 12,857); or 6) data on key covariates were missing from their information (n = 242). To ensure the temporal relationship between exposure and outcome, and to reduce the possibility that depression would affect the likelihood of injury in the following rounds, subjects with a previous history of depression (n = 1,433) at Round 1 and/or concurrent depression at baseline (Round 2, n = 548) were excluded. Finally, respondents who had reported an injury at Round 1 (n = 3,522) were also excluded, to avoid residual confounding by injury-prone characteristics at baseline. The remaining 35,155 subjects comprised the analytic sample.

### Measures

The main predictor in this study was injury at baseline, which was determined from medical condition files using responses to the question of whether “the medical condition they experienced during the 4 or 5 months since the previous interview” was the result of an accident or injury. If the injury happened while the person was at work, it was identified as an occupational injury and ICD-9 codes were used to categorize the injured body region and type of injury based on the Barell classification matrix [11]. Injury severity score was calculated using the Abbreviated Injury Scale with ICD-9 code and the self-perceived overall health impact of the injury. Musculoskeletal disorders encompassed sprains, strains, and dislocations (ICD-9 codes 830–848) and diseases of the musculoskeletal system and connective tissue (codes 710–739). The most recent and severe injury was selected if the respondent reported multiple injury conditions in the preceding 4–5 months. Multiple injury episodes per person, number of healthcare utilizations, and duration of treatment for each injury condition were calculated.

The primary outcome variable in this study was depression incidence at rounds 3 to 5 of the survey. Depression was identified using the ICD-9 codes 296.2 (major depression, single episode) and 311 (depressive disorder, not elsewhere classified). At each interview round, information about depression was collected regarding healthcare utilization, such as prescribed antidepressants, hospital inpatient services, outpatient services, and emergency department services. Analysis was confined to the first reported depression episode for each respondent across the five rounds, because treatment often continued in the following rounds. Later episodes of depression in the same individual were regarded as a continuing treatment or recurrent episode. Individuals experiencing chronic depression that had occurred before the first round of each panel were excluded. Incident cases of depression were defined as those who had first reported depression at rounds 3, 4, or 5.

A comorbidity score was calculated based on D’Hoore’s [12] implementation of the Charlson Comorbidity Index. Occupation was defined using a condensed occupation code, based on the 2003 Census Industry and Occupation Coding scheme, which was collapsed into four occupational groups: white-collar (management, professional, sales, office and administration-related occupations), service, farming (farming, fishing, and forestry occupations), and blue-collar (construction, extraction, and maintenance; and production, transportation and material moving). Based on the risk factors for depression, injury, or both as reported in the literature [2,3,13], five types of potential confounding covariates measured at rounds 1 or 2 were considered in the analysis: sociodemographic factors (age, sex, race, education, marital status, family income level), job-related factors (occupation, company size, self-employment, job tenure, overtime work, work status), medical factors (co-morbidity, activity limitation, self-rated physical and mental health, number of health care events per each condition), health behaviors (current smoking, alcohol or substance abuse problem, exercise, obesity), access to healthcare (insurance coverage, regular visits to a particular doctor or health center), and any cognitive function impairment, such as experiencing confusion or memory loss, having problems making decisions, or requiring supervision for their own safety (yes *vs*. no).

### Data analysis

The distributions of major demographic characteristics and work-related variables among workers with no injury, occupational injury, and non-occupational injury were compared using a chi-squared test. The incidence rates of depression were calculated for persons with injury and compared with the incidence rates among those without injury. A discrete time-proportional odds model [14] was used to estimate the likelihood of an individual developing depression during rounds 3 through 5 based on the experience of the baseline injury condition.

In the univariate analysis, the crude association between baseline injury and depression at follow-up was assessed. In the multivariate analysis, the full logistic regression model included injury and all other variables with a *p*-value < 0.2 in the univariate analysis. Variables that did not reach statistical significance at the *p* ≤ 0.05 level in the regression analysis were removed, but were subsequently retained if their removal changed the magnitude of the main effect by more than 10%. The final model included age, sex, race, education, occupation, family income level, marital status, healthcare accessibility, current smoking, obesity, exercise, activity limitation, cognitive function impairment, comorbidity, perceived physical and mental health status, job tenure, working hours per week, work status, and time since injury. No statistically significant interactions were found for these variables.

To explore the mechanisms that could explain the relationship between injury and depression, six models were tested. Model 1 (base) included the terms age, sex, and time. Model 2 additionally adjusted for race/ethnicity, education, marital status, family income, and health care accessibility. Model 3 added work-related factors such as occupational group, job tenure, number of working hours per week, and work status. Model 4 added smoking, alcohol or substance abuse disorder, exercise, and obesity. Model 5 added activity limitation because of a chronic medical condition, cognitive function impairment, and comorbidity. Model 6 added self-rated physical and mental health status. To evaluate the contribution that each set of risk factors made to the association between injury and depression, the odds ratios (ORs) and 95% confidence intervals (CIs) were calculated for each Model to determine the excess risk.

The initial analysis was carried out separately for men and women. However, the pattern of associations was similar for both sexes and the interaction was not statistically significant, so the results were not reported separately. All analyses were performed using SAS 9.2 (SAS Institute, Cary, NC, USA). A statistically significant association between an exposure and the outcome was declared at a *p*-value < 0.05.

## Results

### Characteristics of the study population

Selected demographic characteristics of the study population at baseline are summarized in Table 1 according to injury group. Injured workers shared several characteristics regardless of whether or not their injury happened at the workplace: they were likely to be unmarried, had greater activity limitation, and had poor perceived physical health status. Several differences existed between workers with occupational and non-occupational injury. Those with workplace injuries were more likely to be male, white, had less education and a lower level of family income; they were also less likely to be insured, and had poor access to healthcare (*p* < 0.01). These workers tended to exercise less, to smoke, and to be more obese at baseline. Conversely, workers with non-occupational injuries tended to be female, never married, had better access to healthcare, and had a higher level of cognitive function impairment than workers with occupational injuries (*p* < 0.01). During follow-up, 1,264 workers experienced depression, with an incidence rate of 3.1% for non-injured workers, 4.7% for workers injured outside the workplace, and 5.5% for workers injured at work.

**Table 1:** Characteristics of the 35,155 respondents at baseline and incidence rate of depression at follow-up

	**No injury (n = 32,544)**	**Non-occupational injury (n = 1,707)**	**Occupational injury (n = 904)**
**Selected characteristics**	**n (%)**	**n (%)**	**n (%)**
Sex (%)*			
Men	16,764 (51.5)	881 (51.6)	598 (66.2)
Women	15,780 (48.5)	825 (48.4)	306 (33.8)
**Mean age (years)**	38.9 (12.0)	38.5 (12.1)	39.5 (11.5)
**Race**			
White	22,691 (69.7)	1,210 (70.9)	644 (71.2)
Black	4,164 (12.8)	211 (12.4)	97 (10.7)
Other	5,689 (17.5)	286 (16.7)	163 (18.0)
Education*			
Less than high school	7,490 (23.0)	480 (28.1)	255 (28.2)
High school graduate	15,009 (46.1)	793 (46.5)	455 (50.3)
College or more	7,639 (23.5)	291 (17.0)	123 (13.6)
Other degree	2,406 (7.4)	143 (8.4)	71 (7.9)
Marital status*			
Married	19,481 (59.8)	921 (53.9)	517 (57.2)
Never married	8,635 (26.5)	505 (29.6)	227 (25.1)
Divorced, widowed, separated	4,428 (13.6)	281 (16.5)	160 (17.7)
**Family income**^┼ ^*			
High	12,578 (38.6)	732 (42.9)	273 (30.2)
Middle	10,878 (33.4)	546 (32.0)	343 (37.9)
Low	9,088 (28.0)	429 (25.1)	288 (31.8)
No usual source of health care*	9,620 (30.0)	389 (22.9)	247 (27.4)
Health insurance coverage*			
Any private	24,335 (74.8)	1,370 (80.3)	643 (71.1)
Public only	1,845 (5.7)	102 (6.0)	54 (6.0)
Uninsured	6,364 (19.5)	235 (13.8)	207 (22.9)
**Exercise**^**‡**^	18,587 (57.1)	1,021 (59.8)	517 (57.2)
**Current smoking**^§ *^	6,420 (19.7)	360 (21.1)	251 (27.8)
**Alcohol or substance abuse problem**			
Obese (BMI ≥ 30) *	8,439 (25.9)	411 (24.1)	292 (32.3)
**Functional activity limitation**^|| ^*	412 (1.3)	38 (2.2)	22 (2.4)
**Cognitive function impairment**	264 (0.8)	24 (1.4)	7 (0.7)
**Comorbidity**^**¶**^	2,940 (9.0)	158 (9.3)	87 (9.6)
Self rated physical health: Poor*	284 (0.9)	19 (1.1)	24 (2.6)
Self rated mental health: Poor*	67 (0.2)	7 (0.4)	4 (0.4)
Incidence of depression at follow-up periods*	1,006 (3.1)	81 (4.7)	50 (5.5)
**Person-round**	130,176	6,828	3,616

* *p* < 0.05.

^┼^Family income: family income as percentage of federal poverty line (high, ≥400% FPL; middle, 200-399% FPL; low, <200% FPL).

^‡^Exercise: having moderate or vigorous physical activity three times per week.

^§^ Current smoker: has smoked at least 100 cigarettes in their lifetime and currently smokes every day or some days.

^||^ Functional activity limitation was defined as having any activity limitation at work or home due to medical condition.

^¶^ Charlson Comorbidity Index ≥ 1.

### Comparison of injury characteristics between workers with occupational and non-occupational injuries

The distribution of injury and work characteristics are presented in Table 2. Individuals with occupational injuries were more likely to be in blue-collar occupations and in smaller companies. They were more likely to work overtime, have lower wage rates, and to have lost more workdays because of injury. Among workers with occupational injuries, a fall was the most common cause of injury, whereas falls, motor-vehicle-related injuries, and sports-related injuries accounted for similar proportions of the non-occupational injuries. Of the injury diagnoses, musculoskeletal disorders accounted for the largest number of both occupational and non-occupational injuries, followed by traumatic complications, open wounds, superficial injuries, and fractures. Occupational injuries in this dataset were more severe than the non-occupational injuries; they had higher severity scores, longer treatment durations, and multiple injury episodes (all *p* < 0.05). Additionally, only 39% of workers with occupational injuries received WC as a source of funding to treat the injury. The rest paid for their treatment themselves, or through other private or public insurance. Those workers who received WC had more severe injuries requiring longer treatment, and tended to be working at larger, and unionized, firms. The incidence of post-injury depression among the workers with occupational injury who were receiving WC was 6.3%, compared with 5.1% of those who were not receiving WC (data not shown).

**Table 2:** Comparison of working and injury characteristics between workers with occupational injury and those with non-occupational injury

	**Non-occupational injury (n = 1,707)**	**Occupational injury (n = 904)**
**Selected characteristics**	**n (%)**	**n (%)**
**Occupational group**^┼ ^*		
White collar	1,017 (59.6)	318 (35.2)
Service	288 (16.9)	164 (18.1)
Farm	6 (0.4)	21 (2.3)
Blue collar	373 (21.9)	388 (42.9)
**Self-employed**	208 (12.2)	99 (10.9)
**Firm size**		
< 10	290 (22.4)	166 (24.4)
10–49	364 (28.2)	205 (30.1)
50–499	432 (33.4)	214 (31.5)
500+	207 (16.0)	95 (14.0)
**Job tenure (yr, mean (SE))**	6.2 (0.18)	6.4 (0.27)
**Hours of working per week (mean (SE))**	38.6 (0.31)	41.7 (0.38)
**Personal annual wage income ($) (mean (SE))**	32,679 (696.7)	28,005 (709.0)
Overtime work (>40 hrs/week)*	450 (26.4)	273 (30.2)
**Part time/seasonal/shift work**	131 (7.7)	54 (6.0)
**Union membership**	200 (13.8)	115 (14.4)
**Cause of injury**		
Fall	343 (33.1)	117 (62.7)
Motor vehicle related	399 (38.5)	57 (20.2)
Sports related	257 (24.8)	0
Other	37 (3.6)	48 (18.4)
**Type of injury: diagnosis category**		
Superficial wound, contusion	148 (8.7)	65 (7.2)
Musculoskeletal (arthropathy, back, sprain/strain)	640 (37.5)	371 (41.0)
Fracture/dislocation	220 (12.9)	59 (6.5)
Crushing, amputation, poisoning, toxic, late effect	69 (4.0)	57 (6.3)
Open wound/internal organ injury	219 (12.8)	125 (13.8)
Traumatic complication, NEC	220 (12.9)	145 (16.0)
Injury severity (ISS) score *		
Minor (ISS 1–8)	956 (56.0)	482 (53.3)
Moderate (ISS 9–15)	537 (31.5)	280 (31.0)
Severe (ISS ≥ 16)	214 (12.5)	142 (15.7)
Injury treatment duration*		
More than one round	342 (20.0)	238 (26.3)
Lost work days *		
More than one day	587 (34.4)	421 (46.6)
No. of injury episodes*		
Multiple	648 (38.0)	383 (42.4)
**Source of payment**		
Workers’ compensation*	N/A	349 (38.6)
Other sources	1,701 (100.0)	555 (61.4)

* *p* < 0.05.

^┼^Occupational group – White collar, Management, business, and financial operations; Professional related; Sales related; and Office and administrative support. Blue collar: Construction, extraction, and maintenance; Production, transportation, and material moving.

*N* may not be 100% due to missing values, and some categories are not shown owing to small number.

SE: standard error.

### Association of injury and depression by work-relatedness

A series of multivariate logistic regression models was used to examine the association between injury and depression after controlling for covariates (Table 3). The covariate that made the strongest contribution to non-occupational injury was cognitive function impairment. Taken together, the final model explained 16% of the depression risk for workers with non-occupational injuries. On the other hand, personal health behaviors such as smoking or exercise explained 15.4% of the excess risk of the association between occupational injury and depression. In the final model, after controlling for all the covariates, occupational injury increased the risk of depression by 72% (OR = 1.72; 95% CI: 1.27–2.32) compared with uninjured workers. The adjusted OR for depression in those with a non-occupational injury was 1.36 (95% CI: 1.07–1.65). Besides occupational and non-occupational injury, female sex, white race, lower income, non-married status, current smoking, obesity, functional activity limitation, cognitive function impairment, perceived health status as poor, part-time working, shorter job tenure, and long working hours were independently associated with higher odds of post-injury depression (data not shown). Workers in the white-collar and service occupations had slightly higher odds of depression (OR = 1.12, 1.10, respectively) compared with those in blue-collar occupations (*p* = 0.06).

**Table 3:** Associations between injury and depression in 35,155 workers according to factors of adjustment

**Factors of adjustment**	**Non-occupational injury**		**% excess risk explained**	**Occupational injury**		**% excess risk explained**
	**Odds ratio**	**95% CI**		**Odds ratio**	**95% CI**	
1 Base ^a^	1.43	1.19–1.80		1.91	1.43–2.56	
2 Socioeconomic status ^b^	1.37	1.10–1.73	14.0	1.78	1.34–2.37	14.3
3 Work-related ^c^	1.40	1.11–1.76	7.0	1.88	1.41–2.52	3.3
4 Lifestyle^d^	1.42	1.13–1.78	2.3	1.77	1.33–2.36	15.4
5 Disability, comorbidity ^e^	1.38	1.10–1.74	11.6	1.88	1.41–2.50	3.3
6 Self-rated health status ^f^	1.40	1.12–1.77	7.0	1.81	1.36–2.42	11.0
7 Full model ^g^	**1.36**	**1.07–1.65**	16.3	**1.72**	**1.27–2.32**	20.9

% excess risk explained is calculated by [(OR _*base*_ – OR _*adj)*_/(OR _*base*_ – 1)]*100.

^a^ adjusted for age, gender, and time.

^b^ adjusted for factors in base model plus race, education, family income, health care accessibility, and marital status.

^c^ adjusted for factors in base model plus occupation, work status (full time *vs.* part time), number of working hours per week, job tenure.

^d^ adjusted for factors in base model plus smoking, obesity, exercise, alcohol or substance abuse problem.

^e^ adjusted for factors in base model plus any activity limitation at work, house, or school due to medical condition, cognitive function impairment, and comorbidity.

^f^ adjusted for factors in base model plus self-rated perceived physical, mental health status.

^g^ Fully adjusted using risk factors from all models.

The impact of a non-occupational injury on subsequent depression appeared to remain steady up to 1 year after the injury (Table 4). In contrast, the effect of occupational injury on depression increased with time after the injury. One year after the injury, a worker who had experienced a workplace injury at baseline was 2.18 times more likely to be depressed than one who had no injury, and a worker with a non-occupational injury was 1.48 times more likely to be depressed than a non-injured worker. The stronger effect of occupational injury did not decrease after controlling for injury severity and other covariates. Compared with the minor injury groups, there were significantly increased odds of depression in workers with moderate-to-severe occupational injury (OR = 2.37, 95% CI: 1.46–3.84) or non-occupational injury (OR = 1.52, 95% CI: 1.03–2.25). WC as a source of medical payment was associated with 33% higher odds of developing depression.

**Table 4:** Final model of depression by time, injury severity, and workers’ compensation status in 35,155 workers

	**Non-occupational injury**		**Occupational injury**	
	**Odds ratio**	**95% CI**	**Odds ratio**	**95% CI**
**Time**				
1 round after an injury	1.10	0.74–1.62	1.36	0.82–2.25
2 rounds after an injury	1.65	1.14–2.38	1.94	1.21–3.11
3 rounds after an injury	1.48	0.99–2.23	2.18	1.32–3.51
**Injury severity**				
Minor	1.0		1.0	
Moderate-to-severe	1.52	1.03–2.25	2.37	1.46–3.84
**Workers’ Compensation**				
No			1.0	
Yes			1.33	1.01–1.74

## Discussion

### Summary of results

This study assessed the differential impact on subsequent depression of occupational injury compared with non-occupational injury. After excluding subjects with previous depression and concurrent depression at baseline, and controlling for relevant covariates including comorbidity, disability, and sociodemographic factors, subjects injured at work showed higher odds of subsequent depression compared with those who had a non-occupational injury. Furthermore, the differential effect increased as the time since injury increased; that is, the longer the time since the injury, the higher the risk of depression, if the injury was occupational. The increased risk of depression among workers with occupational injuries remained substantive after accounting for injury severity and number of treatment episodes. This finding implies that those injured at work may have an increased risk of subsequent depression compared with workers with non-occupational injuries. It may also reflect that the psychosocial aftermath of occupational injury is more complex than non-occupational injury, and is probably related to the longer duration of treatment, lost earnings, and distress involved in litigation for workplace injury. A positive association between post-injury depression and WC insurance claim suggests that the depression risk resulting from occupational injury may not be fully mitigated by WC benefits.

### Limitations and strengths

In interpreting the findings of this study, several limitations must be considered. These include the following: a lack of detailed job attributes and information on other stressful life events; potential recall bias; underestimation of depression in the MEPS; and attrition. First, the dataset did not include information on several potential confounding variables including family history of depression, other stressful life events outside the workplace, detailed job descriptions, and psychosocial conditions in the workplace. This lack of data limited the ability of our analysis to explore the association between occupational injury and depression. Second, the information from the MEPS was self-reported, and thus may introduce recall bias, a common phenomenon in studies using population survey data. However, the intervals between rounds were relatively short at 4–5 months, which should have minimized such bias. The self-reported medical conditions in the MEPS were verified by the Medical Provider Component, a supplemental survey of respondents’ medical providers and pharmacies. The error rate for coding medical conditions based on the ICD-9 code was reported as not exceeding 2.5% on verification. Therefore, it is unlikely that our results were biased by the self-reporting of medical conditions on MEPS. Third, the final sample included a relatively small number of target events (i.e., depression after an injury) despite the large number of people in the full dataset. This limitation may result from inclusion of mainly healthy workers [15], because individuals with depression may be less likely to be in the labor force. Selection bias resulted in relatively small subgroups in the injury categories, likely limiting the analytic power of the study. Indeed, our results from subgroup analyses of occupation, or level of injury severity did not yield statistical significance. Finally, the attrition at the MEPS may not be random. The initial response rate on the MEPS was over 85%, but 30% of respondents had been lost by the fifth round. Thus, attrition could lead to a potential bias; however, the Agency for Healthcare Research and Quality reported that it had no evidence of potential non-response bias attributable to survey attrition on resultant national estimates of healthcare cost [16].

Limitations aside, this study differs from previous research in several ways: it had a longitudinal design; it included a nationally representative sample of the working population; it focused on the differential impact of occupational injury; it differentiated pre-existing depression and post-traumatic stress disorder from post-injury depression; and it controlled for comorbidity. First, by using longitudinal data and by excluding cases at baseline with pre-existing depression among the injured group, this study was able to establish the temporality of the association between injury and depression. Second, use of a representative sample of the working population should allow extrapolation to the general population. Third, this study focused on the impact of occupational injury on depression so it was able to differentiate between the psychosocial consequences of occupational and non-occupational injury. Fourth, using the information for all medically treated or related health conditions, we were able to adjust for comorbidity, physical disability, and other psychiatric conditions associated with increased risk of depression in the final model. Comorbid conditions and physical disability are known to be risk factors for depression as well as injury. By adjusting for these, this study reduced the potential bias from reverse causality in the estimate of risk of depression.

### Prevalence and risk factors of post-injury depression

Our findings were consistent with previous reports of increased risk of developing depression after an experience of traumatic injury. In the general population, depression morbidity after a traumatic injury was reported to range from 2% to 35% at 12 months [17,18]. A few studies [5,6,9] have examined the association between occupational injury and depression. Keogh [6] found that 31% of workers receiving WC reported depression after a musculoskeletal injury in the upper extremities. A recent study based on Canadian WC claims data reported increased levels of depressive symptoms following a work-related musculoskeletal injury, with a prevalence of 42.9% and 26.5% at 1 month and 6 months, respectively [9]. The incidence of depression among workers in this study appeared to be slightly lower than in other studies, although the diversity of measures of depressive symptoms and study populations make it difficult to directly compare our results with others. Although this study sample was restricted to workers without depression at Round 1 and Round 2, there is a possibility that injured workers at Round 2 could have had depression in the intervening period between the two rounds, but did not seek treatment until a later date after injury. This might affect the result by overestimating the incidence of depression after an injury.

Six risk factors were identified for depression following exposure to trauma, including some previously reported [19,20]: female sex; white race; marital status of divorced, widowed, or separated; low income; lack of healthcare access; and functional activity limitation. The most prominent independent risk factors for post-injury depression were self-perceived poor physical and mental health status, functional activity limitation, and cognitive function impairment. This association suggests that a combination of disability and perceived poor health may mediate the pathway between injury occurrence and subsequent depression. Among work-related factors, union membership, long working hours, and small-sized companies also showed a positive association in the relationship between injury and subsequent depression.

Researchers have found that individuals who have low socioeconomic status or who are economically disadvantaged have an increased risk of both injury [21] and depression [2]. In the same manner, workers with lower socioeconomic status are more likely to become injured at the workplace and to become depressed, compared with individuals with higher socioeconomic status. Our results were similar to those of previous studies, which found that injured workers with lower family income had a higher risk of depression across all models, that the risk was more predictive in female workers, while education had little association with non-fatal injuries [21,22].

### Potential mechanism

Our findings indicate that workers with occupational injury are at greater risk of post-injury depression than those with non-occupational injury. A question may be raised here: is the occupational *vs*. non-occupational difference in the risk of depression the result of more severe injuries at work and/or other factors in the potential pathway to depression? Studies have consistently documented the short-term effects of recent stressful life events on episodes of depression [23-27], and injury may play a role as one type of adverse life event. What, then, makes workers with occupational injuries suffer more depression compared with those who have non-occupational injuries? Injury severity or physical disability may not fully explain the observed association.

Previous research on injury and depression provides two potential explanations for the observed higher risk of depression after workplace injury than after non-workplace injury. First, occupational injury may increase the risk of depression by decreasing socioeconomic status and quality of life. Occupational injury may be more severe, and the increased impairment, coupled with unemployment, makes it more likely that injured workers will suffer from depression, compared with those injured elsewhere [6,24]. Our study found a positive relationship between injury severity and post-injury depression, and this effect was stronger for occupational injury than non-occupational injury (Table 3). Second, these emotional consequences may result from the burden of dealing with the complications of the injury or with the WC system [6,24,27-32]. For instance, injured workers have to go through WC to get a benefit, which often entails proving occupational causation and facing the unwillingness of both employers and WC insurers to accept responsibility for the injury. Delays in medical treatment and legal disputes are common experiences for individuals with work-related injuries. Additionally, few WC insurance policies adequately address mental health consequences in their coverage of the rehabilitation of injured workers, leaving individuals to deal with these issues on their own. Our finding that WC covered only 39% of the occupational injury cases suggests that the financial burden of treating an injury could be more of a challenge for workers injured at work than for those injured elsewhere. In the Canadian study, the experience of litigation substantially explained the level of depression in workers who had suffered mild-to-moderate traumatic brain injury with perceived combined stress and pain [20]. In our study population, occupational injury was more severe than non-occupational injury, and although we adjusted for injury severity, there might be a residual confounding from injury severity and from unfavorable working conditions after the injury. Certain job attributes or psychosocial situations at the workplace may also confound or mediate the association [33]. For example, jobs that combine high demand and low control are known to increase the risk of both depression and occupational injury. Long working hours can coincide with high job demand and excessive workload [34], both of which may be risk factors for both injury and depression.

Taken together, these findings support the hypothesis that the effect of occupational injury on depression could be mediated by the financial burden of treating the injury or the difficulties involved in dealing with WC claims. Occupational injuries tended to have higher severity and longer treatment duration than non-occupational injuries, and among workers injured at work, those who received a WC benefit showed a higher likelihood of post-injury depression than non-recipients. This may indicate that distress in dealing with the WC process is part of the causal pathway from injury to depression, in addition to the financial burden people face during longer treatment periods.

### Differential patterns in the association between injury and depression over time by work-relatedness

This study found that the differential effect of occupational injury compared with non-occupational injury on depression increased with time after injury. While most previous studies reported that the depressive impact of a stressful life event occurred shortly after the event, and decreased over time [1], this finding implied that occupational injury may have persistent stressors that occur in the aftermath of injury. These stressors provide unique challenges: the longer the treatment process continues, the more adverse the impact of occupational injury on depression, compared with that of non-occupational injury. Receiving a WC payment may serve as a proxy for unobserved characteristics of occupationally injured workers: workers may become eligible for WC coverage through more severe injury, or by working in a larger company that can provide the WC benefit. Those workers who had WC to cover their medical treatment showed a higher risk of depression compared with those not paid by WC. Thus receiving the WC benefit may not in it itself indicate better treatment, or protection from the adverse consequences of occupational injury. Rather, WC coverage may reflect more severe injury, more expensive treatment, a longer duration of disability, and the difficult process of dealing with the WC system.

## Conclusions

This study found that workers with occupational injury were more likely to become depressed than those with non-occupational injury. The psychosocial consequences of occupational injury, including depression, deserve further exploration to adequately support those injured at work. The findings emphasize a need to develop strategies to reduce the burden of post-injury depression by preventing occupational injury itself, and by providing adequate treatment to reduce the long-term psychosocial consequences of occupational injury.

## Abbreviations

CI: Confidence interval; MEPS: Medical expenditure panel survey; OR: Odds ratio; WC: Workers’ compensation.

## Competing interests

The authors declare that they have no competing interests.

## Authors’ contributions

J Kim planned the study, performed all statistical analysis, and wrote the paper.

## Pre-publication history

The pre-publication history for this paper can be accessed here:

http://www.biomedcentral.com/1471-2458/13/303/prepub
